# What evidence exists on the impact of sustainability initiatives on smallholder engagement in sustainable palm oil practices in Southeast Asia: a systematic map protocol

**DOI:** 10.1186/s13750-022-00283-x

**Published:** 2022-09-01

**Authors:** Jia Yen Lai, Dyah Ita Mardiyaningsih, Faris Rahmadian, Nurfatin Hamzah

**Affiliations:** 1 School of Arts and Social Science, Monash University Malaysia, Subang Jaya, Malaysia; 2 Center for Agriculture and Rural Development Studies (CARDS), Bogor Agricultural University, Bogor, Indonesia; 3 Public Administration and Policy Group, Department of Social Sciences, Wageningen University, Wageningen, Netherlands; 4 Faculty of Science and Marine Environment, Universiti Malaysia Terengganu, Kuala Terengganu, Malaysia

**Keywords:** Land-use governance, Forest-risk commodity, Sustainable supply chain, Voluntary certification

## Abstract

**Background:**

Smallholding plantations represent approximately 40% of the total palm oil plantation area globally. For any certifications, standards, and other instruments to achieve more ethical and sustainable palm oil supply chains, it is essential to improve smallholder engagement in the schemes. A large body of research has built up our understanding of the challenges of engaging smallholders in sustainability initiatives in various sites and countries. A broad systematic understanding of how different types of sustainability initiatives can support or restrict smallholders from access to market and different resources and under which economic and social conditions are not yet developed. This systematic map aims to identify, map, and describe the body of evidence that exists on the positive and negative impacts of sustainability initiatives on smallholder engagement in palm oil practices in Southeast Asia. The findings are expected to inform policies and practices on smallholder engagement in sustainable palm oil supply chains and identify evidence gaps where future primary studies and evidence syntheses can contribute.

**Methods:**

We will develop a guiding framework of interventions through other works on supply chain instruments. We will then construct a test library of 39 items through field expert consultations and snowballing using literature search algorithms. The search will cover four publication databases, five bibliographic databases, and 13 topical and organizational websites. We will search for existing evidence syntheses and primary research studies in Southeast Asia countries published between 2008 and 2021. This systematic map will only include English language articles due to our limited capacity. We will screen the search results at the title/abstract and the full-text levels. Numbers of included/excluded items and reasons for exclusion will be noted and visualized via a ROSES flow diagram. We will develop a data extraction form for assessing data useful for reporting current trends of smallholder engagement in sustainable palm oil initiatives. A random sample of 20% of the included articles will be assessed for validity using Joanne Briggs Institute’s critical appraisal checklist. We will then organize and summarize the data according to the defined PICO.

**Supplementary Information:**

The online version contains supplementary material available at 10.1186/s13750-022-00283-x.

## Background

Palm oil is a plantation crop that continues to raise controversy in the global forest-risk commodity market. Land conversion to palm oil plantations remains the primary driver of deforestation and forest degradation in tropical countries, particularly Indonesia and Malaysia, the two largest palm oil producing countries globally [1]. Interlinked social conflicts, including corruption, contested land tenure, and labour exploitation, are persistent issues in the palm oil supply chains [2–4]. In contrast, governments and companies engaged in palm oil businesses have argued that the productivity and efficiency of palm oil per hectare of land is higher than that of other vegetable oils, which may contribute to lower pressure on land and biodiversity than other vegetable oil productions [5]. Palm oil advocates also commonly highlight the (potential) advantages of the palm oil economy in improving rural livelihoods and reducing poverty. However, these expectations and arguments are often met with mixed evidence on the ground [6, 7].

In response to growing awareness of sustainable and ethical supply chains [8], especially from the palm oil consuming countries and individuals in the Global North, numerous sustainability standards and certifications have been created for palm oil production. However, expensive and techno-centric certification processes have created barriers to engaging smallholders in sustainability initiatives [9, 10]. Notably, disengagement from any sustainability initiatives does not necessarily mean that smallholder practices are unethical or unsustainable. But the fact that such sustainability initiatives have become the central tools for market access in palm oil business [11] can restrict smallholders from equal business opportunities and associated economic incentives, which are important for improving livelihoods and incomes [12]. Smallholders represent approximately 40% of the total palm oil plantation area globally [13]. Improving smallholder engagement is essential for achieving poverty eradication and sustainable palm oil supply chains.

A considerable amount of evidence has demonstrated the challenges of engaging palm oil smallholders in sustainability initiatives. Less is known about how sustainability initiatives can be altered to serve smallholders’ needs better and the conditions under which smallholders can be attracted to or backed off on these initiatives. A few evidence syntheses have covered the role of independent farmers in palm oil supply chains [14–18], focusing on any one type of sustainability initiative or in a single country. A broad systematic understanding of how different types of sustainability initiatives can support or restrict smallholders from access to market and different resources and under which economic and social conditions are not yet developed. This systematic map aims to address this gap by consolidating existing evidence on smallholder engagement in various types of palm oil sustainability standards, certifications, regulations, and other supply chains instruments, identifying engagement conditions associated with specific initiatives, and producing results useful for researchers, practitioners and policymakers engaged in the palm oil certification processes.

## Objective of the review

This systematic map aims to identify, map and describe the evidence on the impact of sustainability initiatives on smallholder engagement in the palm oil practices in Southeast Asia. We are particularly interested in the social and environmental outcomes associated with smallholder engagement in sustainable land use. This exercise will cover sustainability initiatives created and applied by the public and private sector actors, including governments, NGOs, and corporations.

The authors of this protocol initiated and formulated the research questions and the scope of this evidence synthesis. In refining the objective and scope, we consulted several field experts and environmental and development practitioners involved in topics of smallholder engagement in palm oil production in Southeast Asia between February and June 2021. Our systematic map will present a timely perspective on the state of evidence covering various palm oil sustainability initiatives (see “Sustainability initiatives in palm oil supply chains” section below). The findings are expected to inform policies and practices on smallholder engagement in sustainable palm oil supply chains. It will also highlight evidence gaps where future primary studies and evidence syntheses can contribute.

This systematic map will address the following primary research question:

What evidence exists on the impact of sustainability initiatives on smallholder engagement in the palm oil practices in Southeast Asia?

Using the resulting evidence base, we seek to answer the following set of secondary research questions:

1. Which sustainability initiatives are employed for promoting smallholder engagement?
2. According to the study, how does the employed initiative affect smallholder engagement?
3. According to the study, is the employed initiative useful for promoting smallholder engagement, in what aspects, and to what extent (if applicable)?

We define the PICO of this systematic map as:

***Population***

Smallholding oil palm growers, including individuals and their households, in any Southeast Asia country.

***Intervention***

Application of one or more of the defined palm oil sustainability initiatives (see Table 1).

**Table 1:** Sustainability Initiatives in Palm Oil Supply Chains

Type of Initiative	Main actor	Example
Voluntary Sustainability Standards	Companies	RSPO
Internal code of conduct	NGOs	TRASE Finance
Public–private partnership	Companies, NGOs, governments (national or sub-national)	High Carbon Stock (HCS) and High Conservation Value (HCV) standards
Due diligence	Governments	Strategic Sustainable Commodity Action Plan
Multi-stakeholder platform	Companies, NGOs, governments (national or sub-national)	Green Growth Compacts
Jurisdictional approach	Governments	ISPO, MSPO

***Comparator***

Temporal (before/after the adoption of any initiative), spatial (between different sites), or between groups (control/intervention, socioeconomic, gender, racial/ethnic).

***Outcome***

Positive, negative, or neutral effects of smallholder engagement in sustainability initiatives applications. We have referred to the RSPO’s Smallholder Strategy and Accountability Framework initiative’s Smallholder Inclusion in Ethical Supply Chains, and developed four types of engagement to guide the evaluation:

1. Increased participation of smallholders in plantation planning, execution, and/or management (e.g., consultation, capacity training, partnership, communication strategy, and outreach, etc.);
2. Improvement in smallholder livelihood and/or self-sufficiency (e.g., poverty, productivity, risk management, etc.);
3. Improved market access for smallholders (e.g., profitability, financing, marketing, etc.); and
4. Increased tailoring approaches to smallholder inclusion (prioritization of smallholder’s needs).

## Sustainability initiatives in palm oil supply chains

Palm oil sustainability initiatives identified and categorized in our study reflect existing frameworks developed by other works on supply chain instruments [19–22] and an updated review on 2021 [23]. These frameworks document the efforts aiming to achieve zero-deforestation commodity supply chains following socially and environmentally feasible standards. Specifically, we draw insights on sustainability initiatives for palm oil supply chains from these sources to define the intervention in this systematic map.

We have identified six types of initiatives available in the governance of sustainable palm oil supply chains: Voluntary Sustainability Standards (VSS), internal code of conduct, public–private partnership, due diligence, multi-stakeholder platform, and jurisdictional approach (see Table 1). These initiatives are classified by the principal actors that design and utilize them, namely corporations, governments, or non-governmental organizations [23].

VSS are market-driven mechanisms, mainly created and promoted by palm oil producers and companies, for addressing social and environmental issues in the production, processing, and trade of palm oil products [24]. The certification of Roundtables on Sustainable Palm Oil (RSPO) has been the most widely recognized VSS in the governance of sustainable palm oil supply chains [5]. High implementation costs and complex sustainability and legal standards applied in the producing and consuming regions/countries, however, pose persistent challenges to smallholder engagement in VSS [25].

Internal codes of conduct are often complemented to govern the transparency in the palm oil supply chains alongside VSS [20, 26]. For example, Transparency for Sustainable Economics (TRASE) has sought to evaluate the financing of traders exporting Indonesian palm oil [27].

Public–private partnerships, mainly linked to zero-deforestation commitments, are also increasingly adopted by palm oil companies [28]. High Carbon Stock (HCS) and High Conservation Value (HCV) standards [29], Peatland Moratorium in Indonesia [30], Accountability Framework initiative (AFi) [31] are some prominent pledges guiding companies on sustainable palm oil supply chains.

The role of importing countries in achieving sustainable supply chains is increasingly recognized, resulting in a growing emphasis on bilateral and multilateral collaborations between producer and consumer countries. Some importing countries have also created due diligence and other policy instruments to combat imported deforestation linked to palm oil [32]. For example, the UK’s Strategic Sustainable Commodity Action Plan [33] and France’s National Strategy to Combat Imported Deforestation [34].

A new generation of initiatives, also led by collaborative efforts, usually involves policy instruments, state regulations, and private sector tools (including codes of conduct). These multi-stakeholder platforms usually highlight the “hybrid” or “multi-partner” forms of sustainable governance [35]. While many multi-stakeholder platforms remain exploratory, some have constituted partnerships for setting up companies’ good practices in conjunction with regional land-use planning and service-provision schemes, e.g., Green Growth Compacts in East Kalimantan, Indonesia [36].

Finally, jurisdictional approaches are broadly defined as wall-to-wall frameworks that align governments, businesses, NGOs, and local stakeholders in specific administrative jurisdictions with common interests in land-use governance [37]. These approaches usually co-existed with the implementation of other initiatives, especially VSS, but they feature a high level of government intervention to manage environmental and economic trade-offs more effectively [21]. The implementations of jurisdictional approaches are also often accompanied by adjustments in incentive systems seeking alignment to shared sustainability goals between governments and public sector actors. The Indonesian Sustainable Palm Oil (ISPO) and the Malaysian Sustainable Palm Oil (MSPO) are two major instruments under this category.

Although these approaches emphasize the goal of zero-deforestation, they have increasingly recognized the implications of socio-economic factors, including smallholder engagement, on achieving sustainable palm oil supply chains. While we expect RSPO, ISPO, and MSPO to be the prominent interventions in this field of study, we include all six types of initiatives in our investigation to document the state of evidence more comprehensively.

## Methods

We will identify evidence from existing evidence syntheses and primary research studies in any Southeast Asia country. A comprehensive search for peer-reviewed literature and other publicly available studies will be conducted using multiple sources of knowledge. The systematic map will cover the publications between 2008 and 2021. We have chosen this temporal scope because the RSPO Supply Chain Certification Systems (SCCS), the first widely-acknowledged palm oil sustainability initiative, was finalized in August 2008 [41].

Three components drive our search strategy: (a) constructing a test library, (b) establishing a search string that captures the enabling or disabling factors, and (c) developing the scope of the search and eligibility criteria.

### Constructing test library

**Fig. 1:**
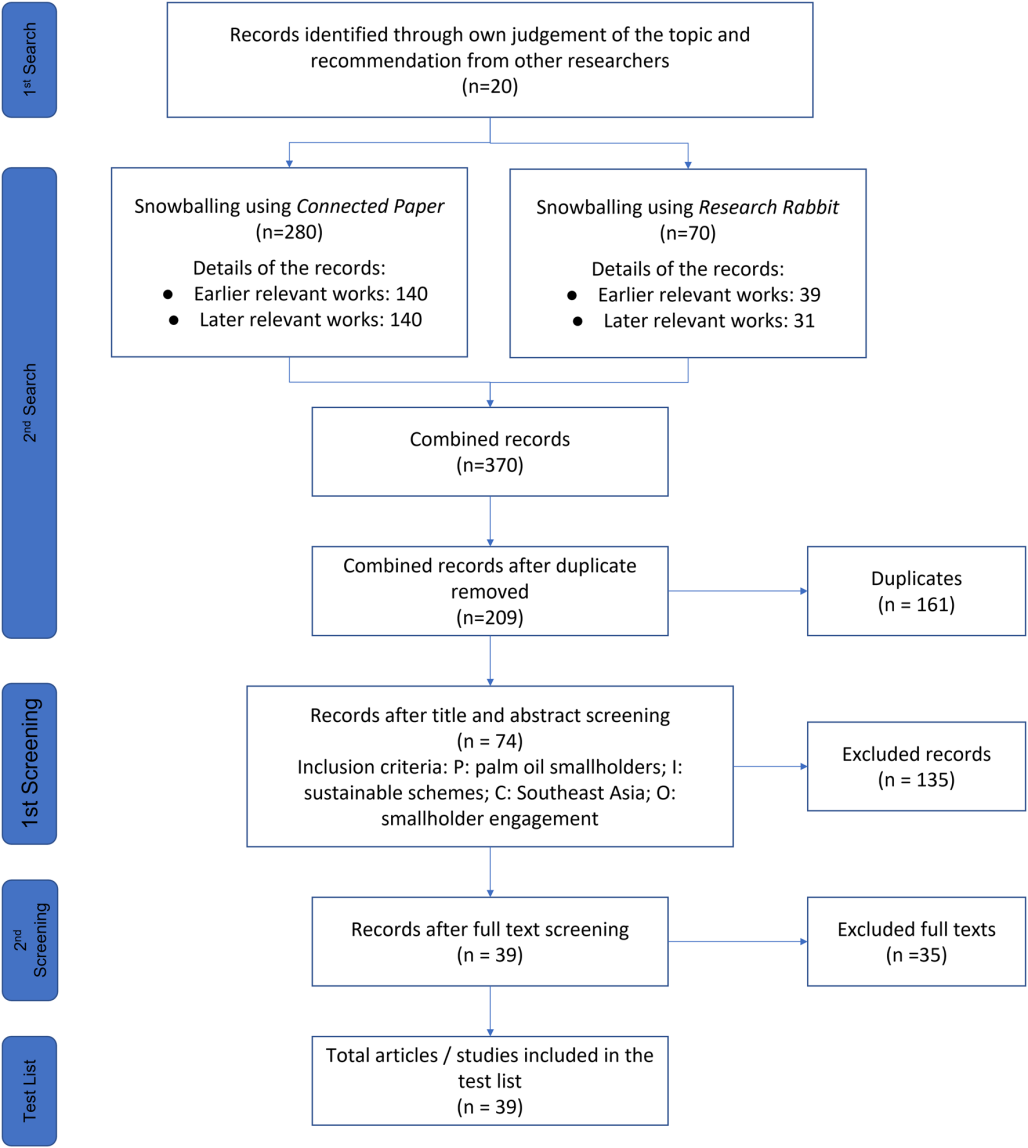
Flow chart of test library construction

The authors and the field experts consulted have contributed to an initial list of 20 publications to the test library. We have snowballed relevant publications by importing the initial list into two literature search applications: *Connected Papers* [38] and *ResearchRabbit* [39]. See Fig. 1 Flow chart of test library construction.

The algorithm of Connected Papers tracks the most relevant prior and derivative works that followed the 20 input papers. The relevant papers suggested by the algorithm are selected according to the similarity to the origin papers. Any two papers with highly overlapping citations and references are presumed to have a higher chance of treating a related subject matter. The database of Connected Papers is connected to the Semantic Scholar Paper Corpus [40].

ResearchRabbit collects relevant earlier and later works, and by manually fine-tuning the collection, the algorithm generates targeted suggestions to a related subject matter. Although it is unclear how the RR algorithm is constructed and what databases are used, the impact of lacking such information is limited because we will screen all suggested papers according to the predefined criteria. We consider RR useful for searching relevant papers and therefore decided to include this algorithm in the construction of the test library. See Additional file 1: Test Library.

### Searching for articles

#### Search terms and languages

We compiled an initial set of English search terms relevant to different components of the research question in consultation with a librarian. We then ran the initial set of keywords on ProQuest Thesaurus to collect other suggested keywords (Additional file 2). Only English articles are included in this study due to the capacity of the review team.

#### Search strings

We have created the search strings using the search terms collected in the process mentioned above.

Population terms ("smallhold*" OR "small-hold*" OR "small hold*" OR "individual" OR "scheme" OR "small scale farmer*" OR "land owner*")

AND

*Management terms (*“palm oil” OR “oil palm” OR “elaeis guineensis”)

AND

*Outcome terms (*“engage*” OR “engaging” OR “empower*” OR “job*” OR “employ*” OR “skill*” OR “livelihood*” OR “income*” OR “produc*” OR “wealth*” OR “wellbeing*” OR “well being*” OR “market” OR “access” OR “security” OR “vulnerabilit*” OR “yield*” OR “inclusiv*” OR “capital” OR “perception*” OR “preference*” OR “awareness” OR “equity” OR “right*” OR “participat*” OR “conflict*” OR “justice*” OR “communit*” OR “involvement” OR “benefit*” OR “compensation” OR “trade off*”)

AND

*Intervention terms (*“sustainab*” OR “voluntary” OR “scheme*” OR “standard*” OR “certif*” OR “partnership*” OR “agreement*” OR “innovati*” OR “initiative*” OR “incentive*” OR “regulat*” OR “framework*” OR “contract*” OR “RSPO” OR “invest*” OR “collaborat*” OR “pledge*” OR “assessment*” OR “multi-stakeholder*” OR “cooperate*” OR “best practice*”)

#### Estimating the comprehensiveness of the search

We have conducted a scoping exercise on Web of Science to examine the sensitivity of different term combinations and to refine the search string. We have tested the search string by running it against the test library in three sources: Web of Science, Scopus, and Google Scholar. 87% or 34 of 39 studies are captured by the search string and by more than one source (Additional file 2). We have intentionally included 8 working reports in the test library to check if Google Scholar can capture grey literature effectively. Only two of the working reports are captured by Google Scholar. We have therefore expanded the search to other organizations’ databases (Table 2).

**Table 2:** List of topical databases

Source	Web URL
The International System for Agricultural Science and Technology (AGRIS)	https://agris.fao.org/agris-search/index.do
Center for International Forestry Research (CIFOR)	https://www.cifor.org/knowledge/
International Food Policy Research Institute (IFPRI)	https://www.ifpri.org/publications
Consortium of International Agricultural Research (CGIAR)	https://cgspace.cgiar.org/
World Agroforestry	https://www.worldagroforestry.org/publications-all
Roundtable on Sustainable Palm Oil (RSPO)	https://www.rspo.org/resources
World Bank	https://openknowledge.worldbank.org/
Tropical Forest Alliance	https://www.tropicalforestalliance.org/en/insights/publications/
Principles for Responsible Investment	https://www.unpri.org/
Strengthening Palm Oil Sustainability in Indonesia (SPOS Indonesia)	https://sposindonesia.org/
Jopeh the Publication of the Malaysian Palm Oil Council (MPOC)	https://www.jopeh.com.my/index.php/jopecommon/issue/all
Sawit Watch	https://sawitwatch.or.id/category/buku/
World Resource Institute (WRI)	https://www.wri.org/resources/type/research-65

#### Publication databases to be searched

To capture an unbiased representation of existing studies, we will search on Web of Science Core Collection, Scopus, and Garuda. WoS and Scopus are commonly used in the searches for environmental-thematic evidence synthesis [24, 42]. Garuda [43] is a local knowledge database that covers 14,311 journals mainly in Indonesia (up to 7th April 2022). By including Garuda in the search, we hope to identify locally published studies that may not be recorded in other international databases.

#### Internet searches and specialist searches to be conducted

We have identified several topical and organization repositories and specialist websites on related topics based on the authors’ experiences, field experts’ suggestions, and searches using combinations of keywords on Google (Table 2). We also find Google Scholar particularly useful in identifying non-indexed but publicly available publications.

#### Supplementary searches

Several relevant evidence syntheses have explored issues pertinent to our research scope [14–18]. We will include those syntheses’ bibliographies in the screening process.

### Reference management

We will use Zotero to record and manage candidate studies identified. Our literature library will be publicized on Zotero Web Library once completed. We will search on the Google Scholar database through the assistive application Harzling’s Publish or Perish [44]. While one needs to save citations one by one on the web interface of Google Scholar, Publish or Perish helps generate the citations in batches, which will enable more efficient and controlled searching exercises.

### Article screening and study eligibility criteria

#### Screening process

We will review search results at the title-abstract and full-text levels to decide on eligibility. In the first stage, the authors will review the titles and abstracts of all papers and exclude the papers that do not meet the eligibility criteria. The included papers will then be reviewed at the full-text level. A screening training will be employed for consistency checking. The review team will collectively perform a calibration exercise on a random sample of 10 studies at the title and abstract level to ensure agreement on the eligibility criteria. Once the team finds alignment on eligibility criteria, the reviewers will independently conduct title and abstract screening. Any candidate article authored by a reviewer will be assigned to the other interest-neutral reviewers. The article will be marked and randomly assigned for a second review where an author/reviewer is unsure about inclusion. Another calibration exercise of another 10 random samples and independent screening at the full-text level will be performed after the title and abstract screening is completed. A double screening at the title and abstract and the full-text levels will be conducted at five percent of the studies. A complete list of included and excluded studies, including exclusion reasons, will be provided in the final report. The lead researcher will crosscheck the excluded articles and the reasons for exclusion prior to the study validity assessment.

#### Eligibility criteria

The inclusion criteria are outlined according to the defined PICO as mentioned above.

##### Eligible populations

We will follow the definition of smallholder set by RSPO [45]: *Smallholders are farmers who grow oil palm, alongside with subsistence crops, where the family provides the majority of labour and the farm provides the principal source of income, and the planted oil palm area is less than 50 hectares.* Smallholders include associated, independent, and schemed growers. Data on smallholders’ gender, ethnicity, and other socioeconomic characteristics will be documented and assessed to understand the distribution of evidence.

##### Eligible interventions

We have defined included interventions in Table 1*.* We will include studies on smallholders’ experiences in engaging (or disengaging) in palm oil sustainability initiatives. Some studies may be recorded in multiple intervention categories if various intervention activities are involved in the studies.

##### Eligible comparators

Included studies should employ a comparator, which indicates the results of change due to the intervention and its implication on smallholder engagement. Valid comparators include but are not limited to temporal, spatial, and between-groups comparators. We intend to cover a wide range of comparators, research methods, and data types to capture the overall state and characteristics of the evidence base.

##### Eligible outcomes

Included studies should evaluate the advantages and disadvantages, benefits and difficulties of smallholder engagement in adopting sustainability initiatives.

##### Eligible types of study design

We will include studies generating primary data. They can be non-experimental, quasi-experimental, and experimental study designs that use quantitative, qualitative, or a combination of data types. All studies should cover at least one Southeast Asia country. We follow the list of ASEAN member states covering Brunei Darussalam, Myanmar (Burma), Cambodia, Indonesia, Lao DPR, Malaysia, Philippines, Singapore, Thailand, and Vietnam. We will also include the two observer states of ASEAN: Papua New Guinea and Timor Leste. Due to our capacity, we will only cover English-language articles.

#### Study validity assessment

We will use Joanne Briggs Institute’s critical appraisal checklist for qualitative, cross-sectional, cohort, economic evaluations, and prevalence studies to evaluate the validity of studies and identify sources of bias. A random sample of 20% of the included articles will be independently assessed for validity by two reviewers. The two reviewers and the lead researcher will then discuss and resolve any concerns or disagreements regarding the samples’ validity. The lead researcher will also be the third reviewer of the sample articles in question. We may refine the checklists to reflect the characteristics of socio-environmental studies more accurately when we test the sample studies. Any changes to the checklists will be explained in the final report.

#### Data coding strategy

Many studies on smallholder engagement have used qualitative approaches due to the nature of interdisciplinary, socio-environmental research. We will seek to evaluate the outcome qualitatively rather than using statistical data. A data coding form will be employed to code data from all included studies. We will document bibliographic information of the study and relevant data to our research scope. Population type, intervention type, comparator details, outcome details, study design, study area, and scale will be recorded (Additional file 3). The research team will conduct full-text assessment training for consistency checking. The training should also help resolve any ambiguities and refine the data extraction form. Five percent of the included studies will be randomly selected for a second review. Any candidate article authored by a reviewer will be assigned to the other interest-neutral reviewers.

This systematic map will highlight issues of smallholder engagement in the palm oil supply chains in Southeast Asia. We expect to identify many studies focused on two major palm oil producing countries, namely Indonesia and Malaysia. A focus on palm oil smallholders may also lead to similar socioeconomic status. Geographical and socioeconomic factors, therefore, will be observed closely to identify effect modifiers or reasons for heterogeneity.

Some potential effect modifiers or reasons for heterogeneity may be as follows:

1. Type of smallholders (independent/schemed, landowner/worker);
2. Socioeconomic status (social class, income, education, etc.)
3. Cultural factors (exposure to market-based systems, farming techniques, etc.);
4. Natural disasters and/or climatic conditions; and
5. Size, age, gender of the study community.

We will identify and record the effect modifiers/reasons during the full-text assessment. Any of such effects on the conclusions will be discussed in the final report.

#### Study mapping and presentation

The data will be organized and summarized according to the defined PICO. The results will present the patterns in the evidence and provide insights and recommendations for policy and research. That includes the identification of evidence gaps and synthesis gaps on any aspects of smallholder engagement in the palm oil supply chains. We may use descriptive statistics to describe the metadata. Figures and tables will be used for presenting evidence clusters and knowledge gaps if needed. The data collected and coded will be presented on spreadsheets. We will publicize the respective metadata spreadsheets with the systematic map. The full list of literature will be uploaded to an open-access reference library.

## Supplementary Information

**Additional file 1. **Test library.

**Additional file 2. **Searching strategy development.

**Additional file 3. **Data coding form.

**Additional file 4. **ROSES form for systematic map protocols.

## Data Availability

All data generated or analyzed during this study are included in this published article and its additional files.
